# Genetic Basis of ACTH-Secreting Adenomas

**DOI:** 10.3390/ijms23126824

**Published:** 2022-06-19

**Authors:** Pietro Locantore, Rosa Maria Paragliola, Gianluca Cera, Roberto Novizio, Ettore Maggio, Vittoria Ramunno, Andrea Corsello, Salvatore Maria Corsello

**Affiliations:** 1 Unit of Endocrinology, Department of Translational Medicine and Surgery, Università Cattolica del Sacro Cuore—Fondazione Policlinico “Gemelli” IRCCS, Largo Gemelli 8, I-00168 Rome, Italy; pietro.locantore@icloud.com (P.L.); gianluca.cera@outlook.com (G.C.); robertonovizio@gmail.com (R.N.); ettoremaggio94@virgilio.it (E.M.); vittoria.ram@hotmail.it (V.R.); andreacorsello92@gmail.com (A.C.); corsello.sm@meridiaroma.it (S.M.C.); 2 Unicamillus, Saint Camillus International University of Medical Sciences, via di S. Alessandro 10, I-00131 Rome, Italy

**Keywords:** Cushing’s disease, ACTH-secreting adenoma, genetic mutations, driver genes, USP8, germline mutations

## Abstract

Cushing’s disease represents 60–70% of all cases of Cushing’s syndrome, presenting with a constellation of clinical features associated with sustained hypercortisolism. Molecular alterations in corticotrope cells lead to the formation of ACTH-secreting adenomas, with subsequent excessive production of endogenous glucocorticoids. In the last few years, many authors have contributed to analyzing the etiopathogenesis and pathophysiology of corticotrope adenomas, which still need to be fully clarified. New molecular modifications such as somatic mutations of *USP8* and other genes have been identified, and several case series and case reports have been published, highlighting new molecular alterations that need to be explored. To investigate the current knowledge of the genetics of ACTH-secreting adenomas, we performed a bibliographic search of the recent scientific literature to identify all pertinent articles. This review presents the most recent updates on somatic and germline mutations underlying Cushing’s disease. The prognostic implications of these mutations, in terms of clinical outcomes and therapeutic scenarios, are still debated. Further research is needed to define the clinical features associated with the different genotypes and potential pharmacological targets.

## 1. Introduction

Cushing’s syndrome (CS) is a complex clinical entity, arising from chronic exposure to excessive levels of glucocorticoids. It can be classified in ACTH-independent CS, resulting from adrenal disease, and ACTH-dependent CS, in the case of the overproduction of ACTH with secondary hypercortisolism. ACTH-dependent causes can be further classified into two groups based on the source of ACTH production: ACTH-secreting pituitary tumors (also termed Cushing’s disease) and ectopic ACTH-production. The clinical features of this syndrome include common physical manifestations such as hypertension, central obesity, hyperglycemia, hyperlipidemia, urolithiasis, and fatigue as well as more specific features that can help in the diagnostic process (i.e., bruising, hirsutism, plethora, proximal muscle weakness, osteopenia/osteoporosis (possibly presenting with bone fractures), and wide purple striae). Mental aspects of this syndrome include intermittent depression, insomnia, and altered memory and concentration. In addition, in the case of Cushing’s disease, local mass effect signs or symptoms may arise in the case of larger pituitary adenomas including headache and visual field defects [1,2].

The diagnosis of CS requires the confirmation of hypercortisolism, which can be conducted with three laboratory tests including urinary free cortisol (24 h urine collection), low-dose dexamethasone suppression test (Liddle 1 or 1 mg overnight test), and late-night salivary cortisol to assess the absolute cortisol levels and the integrity of the circadian rhythm of endogenous glucocorticoids [3]; if available, other tests such as midnight serum cortisol may be used. Once CS has been diagnosed, the plasma ACTH measurement determines whether the patient is affected by an ACTH-independent or ACTH-dependent form. In the case of undetectable ACTH levels, adrenal disease is suspected, and abdominal MRI/CT imaging is recommended. Conversely, with non-suppressed ACTH levels, a high dose dexamethasone suppression test (Liddle 2 or 8 mg overnight test) is performed. An adequate cortisol suppression after this test usually indicates pituitary disease and pituitary MRI is indicated; an inadequate suppression usually indicates an ectopic production, and other tests are required to define the source of ACTH excess (body CT/MRI, octreoscan, or Gallium-68 PET/CT scan). In the case of unclear scenarios, a further test may be indicated such as CRH stimulation test or bilateral inferior petrosal sinus sampling (BIPSS). This diagnostic procedure needs to be carefully implemented and interpreted, especially in pediatric patients and in patients affected by adrenal disease with the suspicion of concomitant Cushing’s syndrome. First, in children, a different frequency of causes of CS can be observed (i.e., ectopic ACTH excess is rare in children vs. adults, while adrenal disease is more frequent than CD in children under the age of 7) and the acceptability and invasiveness of diagnostic tests should be considered (e.g., favoring salivary cortisol) [1,4]. Moreover, in patients affected by adrenocortical carcinoma treated with mitotane, ACTH secretion can be impaired [5] and total serum and urinary cortisol values can be altered, making salivary cortisol a potentially more accurate assay [6].

Treatment of Cushing’s disease mostly relies on pituitary surgery, if possible; second, radiotherapy and/or bilateral adrenalectomy may be needed. Pharmacologic therapies include pituitary targeted molecules such as pasireotide or cabergoline; adrenal-directed drugs such as ketoconazole, mitotane, osilodrostat, metyrapone, and etomidate; and glucocorticoid receptor antagonists such as mifepristone. New therapies are being studied, also thanks to the recently discovered molecular alterations that determine the etiopathogenesis and pathophysiology of this disease. This review aims to present the most recent updates on genetic mutations underlying Cushing’s disease.

## 2. Results

We conducted a search on Pubmed.gov (accessed on 4 April 2022) to identify relevant papers related to the genetic basis of Cushing’s disease. Our search included the following keywords: Cushing’s disease, ACTH-Secreting Pituitary Adenoma, pituitary neoplasms, genetics. We focused our search on more recent articles using filters based on publication dates (2016–2022 and 2021–2022) to collect updated information. 

### 2.1. Somatic Driver Mutations 

#### 2.1.1. USP8

USP8 is one of many deubiquitinase proteins of the USP family. Recent studies have contributed to clarify the USP8 protein function and its alterations in Cushing’s disease. The USP8 protein is constituted by several domains including the catalytic USP domain, which has deubiquitylating activity and can be activated through cleavage. USP8 also contains a WW-like autoinhibitory domain, which interacts with the catalytic domain, impeding its bonding with ubiquitylated proteins, and avoids the activating cleavage of the protein [7]. USP8 is modulated through the binding of 14-3-3 proteins with the WW-like domain, which stabilize it in an inactive conformation [7]. 

In 2015, two different research groups reported the presence of various mutations in exon 14 of the *USP8* gene in the majority of the analyzed corticotrope adenomas. Ma et al. conducted a DNA exome sequencing of 12 fresh tumor samples and the additional Sanger sequencing of DNA samples from 108 other ACTH-secreting adenomas, finding *USP8* mutations in 62.5% adenomas (75/120) [8]; Reincke et al. performed tumor DNA exome sequencing on 10 samples and *USP8*-targeted sequencing on an additional seven samples, finding an overall prevalence of 35% (6/17) [9]. These studies have notably increased our understanding of the molecular pathogenesis of Cushing’s disease. Many other authors have confirmed the presence of the reported mutations since then, and other mutations have been described in several subsequent studies [10,11,12]. Interestingly, *USP8* has also been studied in other tumors and been found to be upregulated in cervical squamous cell carcinoma and lung carcinomas, representing a negative prognostic factor [13], even though its mutations remain specific markers of corticotrope adenomas [14].

Overall, the reported prevalence of *USP8* mutations is 23–62% of corticotrope adenomas. It is of note that a higher prevalence of *USP8* mutations has been reported in Chinese studies [8] compared to European [9,12,15] and American studies [12,16], possibly because of an interplay with genetic and ethnic differences [17,18].

Most of the reported mutations in ACTH-secreting adenomas alter the amino acid sequence in the 14-3-3 binding site and disrupt the USP8 and 14-3-3 bond, thus un-inhibiting USP8 activity because of increased cleavage of the catalytic domain [9] and/or increased spontaneous enzymatic activity even without such cleavage [7]. Other mutations also reduce 14-3-3 binding, not because of altered sequences in the bonding region but because of conformational abnormalities [10].

In cultured ACTH-secreting adenoma cells, USP8 dysregulation is associated with an increased expression of EGFR and other proteins involved in EGF signaling including CMTM8, MAPK15, and E2F1 transcription factor, which contribute to the genesis of corticotrope adenomas and sustain ACTH and POMC secretion [15,19,20,21]. In vitro studies showed increased EGFR activity with ERK1/2 activation, leading to Nur77 binding to the Nur responsive element in the promoter of *POMC* and subsequently increased ACTH secretion [9]. An in vivo model supports the link between EGFR upregulation and corticotrope adenoma formation: Araki et al. developed a transgenic mouse model overexpressing EGFR in corticotrope cells, showing the development of corticotrope adenomas and a Cushing phenotype [21]. These represent actual and potential therapeutic targets: gefitinib has shown promising cytotoxic and/or anti-secretive EGFR-inhibition-mediated effects in vitro and in murine models [14,20,21]; Bexarotene-Lapatinib combined EGFR- and transcription inhibition has been reported as potentially useful in vitro [22]. 

ACTH oversecretion in *USP8*-mutated adenomas may also be explained by the fact that USP8 hyperactivation may directly cause a reduction in POMC and POMC precursors of ubiquitin-mediated degradation; conversely, ACTH is not a target of ubiquitylation as it is synthetized outside the endoplasmic reticulum: its increased secretion in *USP8*-mutated adenomas appears then to be a consequence of increased EGF signaling and POMC production, as shown in corticotrope adenoma primary cultures [15].

**Figure 1:**
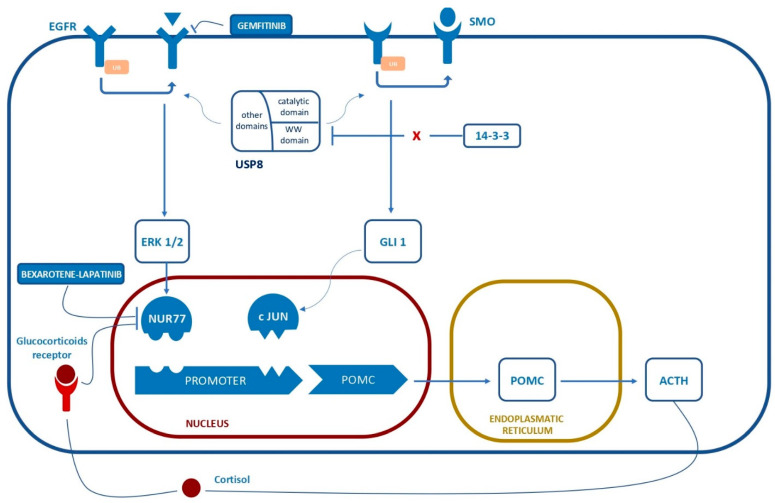
The outline of the relevant pathways involved in ACTH secretion related to the described mutations and potential druggable targets.

*USP8*-mutated corticotrope adenoma cells are more sensitive to corticotrope modulators, namely CRH and dexamethasone, in vitro, compared to *USP8*-wild-type (WT) ACTH-secreting adenomas, with higher levels of CRH and glucocorticoid (NR3C1) receptors [15]. Interestingly, a coexisting mutation of the *RASD1* gene has been reported in a case of *USP8*-mutated adenoma [23]; a murine model supports the hypothesis that *RASD1* is induced by dexamethasone in corticotrope cells and likely plays a role in glucocorticoid-mediated negative feedback functioning as an inhibitor of the CRH signaling pathway [24]. Over- or under-expression of many genes involved in EGFR recycling, cell cycle regulation (e.g., p27 and CABLES1), POMC expression and ACTH secretion (e.g., CREB), glucocorticoid receptor activity (e.g., HSP90), and more general granule sorting and exocytosis have also been found in *USP8*-mutated corticotrope adenomas, primary cultures, and in the murine corticotrope adenoma AtT-20 cell line [15,25]. An increased expression of somatostatin receptor SSTR5 has also been reported in a transcriptome analysis via the RNA sequencing of human ACTH-secreting adenoma cells, with potential therapeutic implications (i.e., possible different sensitivity to pasireotide) [26]. Finally, USP8 has been shown to increase Sonic Hedgehog (SHH) signaling by promoting SMO activity [27], even if the possible implications of this alteration have not been studied in *USP8*-mutated corticotrope adenoma cells to date (Figure 1).

A recent study analyzed the different expression of microRNAs via RNA sequencing and cDNA analysis in formalin-fixed tumor samples; the authors found 68 differentially expressed microRNAs between the *USP8*-mutated and WT ACTH-secreting adenomas, with 400 putative target genes for these miRNAs. Twenty-five of these genes, mainly involved in ubiquitylation processes, were differently expressed between the two groups. Only 12 of the 68 identified miRNAs target these genes [28]. The preliminary findings of these studies suggest that the prominent epigenetic differences between *USP8*-mutated and WT corticotrope adenomas are not caused by the alteration of miRNAs expression.

This constellation of molecular alterations associated with *USP8* mutations has led some authors to recommend categorizing of the mutational status of this gene in ACTH-secreting pituitary adenomas [29]. This may be supported by some case series that report relevant clinical differences in patients with *USP8*-mutated compared to *USP8*-WT adenomas (e.g., higher prevalence in females, smaller size, lower rates of parasellar invasion, better post-surgical outcomes but higher rates of recurrences) [8,9,12,30,31,32,33,34], although with conflicting results [16,18,26,32,33,34]. In a case report, *USP8* mutational status was of aid in diagnosing Cushing’s disease in a patient with dubious immunohistochemical and biochemical features, suggesting a potential diagnostic role for this molecular alteration [35].

*USP8* mutations have also been described in pediatric patients with corticotrope adenomas [32], but a recent study advocates that it may be less frequent than in adult patients [36]. It is possible that other molecular mechanisms prevail in pediatric corticotrope adenomas than in adults. A single case report reported a *USP8* germline mutation in a patient with a syndromic clinical presentation that included Cushing’s disease (see below) [37]. 

#### 2.1.2. Other Driver Mutations

**Figure 2:**
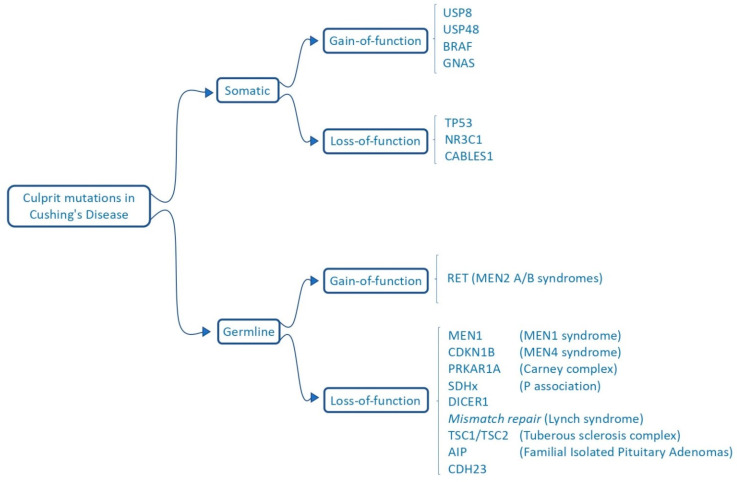
A list of the somatic and germinal mutations reported in ACTH-secreting adenomas.

After the identification of *USP8* mutations, many research groups aimed to identify other potential driver gene mutations that could clarify the tumorigenesis of *USP8*-WT corticotrope adenomas. Many new mutations have been identified, and to date, around 12–28% of ACTH-secreting adenomas have no known mutations [38] (Figure 2). Interestingly, some of these new driver mutations that have been identified in *USP8*-WT tumors are not mutually exclusive one to each other [39]. The following paragraphs focus on the reported driver mutations and their pathophysiological consequences, ordered by prevalence, although the estimated prevalence may be biased by the different methods used in molecular studies (i.e., analysis of specific target genes as opposed to tumoral DNA Next Generation Sequencing; inclusion of *USP8*-WT adenomas only) [38]. In fact, a high variability of such percentages has been observed [39]. Different genetic backgrounds of the studied populations may also explain such heterogeneity among the studied populations [18].

***USP48*** codifies for another deubiquitinase that is linked to increased POMC and ACTH synthesis. This protein shares with USP8 and the other USP deubiquitinases a similar catalytic domain, composed of a “finger–palm–thumb” structure [40]. Two missense mutations of *USP48* involving methionine 415 (i.e., Met415Ile, Met415Val) have been described [41,42]. Interestingly, these mutations directly involve the catalytic domain of the protein rather than other regulatory domains, as seen instead in *USP8* mutations. These modifications constitutively increase the deubiquitinase activity of USP48, leading to the formation of corticotrope adenomas and ACTH hypersecretion [41]. The underlying molecular pathogenic mechanism may lie in the activation of transcriptional factor NF-κB and the subsequent *POMC* promoter activation, as seen in AtT-20 cells [43,44]. Another described mechanism could involve the Sonic Hedgehog (SHH) pathway. CRH signal transduction requires SHH-pathway zinc finger protein Gli1: in fact, CRH stimulus is impaired in vitro in Gli1-silenced AtT-20 cells; USP48 has been shown to deubiquitinate Gli1 and histone H2A, therefore dysregulating CRH signal transduction and cell growth, possibly resulting in adenoma formation with dysregulated ACTH secretion [41]. 

The prevalence of *USP48* mutations greatly varies among the different studies, ranging from 3 to 23% of the analyzed samples [10,39,42]. As for the clinical features associated with these mutations, it has been reported in a case series that *USP48*-mutated corticotrope adenomas (evaluated via exome and Sanger sequencing of fresh and/or formalin-fixed tumor samples) showed a smaller size and higher prevalence among female patients compared with *USP8-/USP48*-WT tumors [41]. In another case series, *USP48* mutations were also associated with an increased risk of cavernous sinus invasion, although in a small sample size [39]. 

***TP53*** codifies for a protein involved in many fundamental cellular processes linked to genome stability and proliferation. Mutations of this tumor suppressor gene have rarely been detected in pituitary ACTH-secreting carcinomas [45] but had not been described in corticotrope adenomas until 2009 [46]. Next Generation Sequencing has allowed researchers to identify other loss-of-function *TP53* mutations (i.e., frameshift/nonsense inactivating mutations or missense copy number loss mutations) in approximately 12.5% of cases of ACTH-secreting adenomas [9,38,42]. Coexisting mutations of both *TP53* alleles have been described; alternatively, a mutation of one copy of *TP53* and either loss of heterozygosity or a mutation of another gene involved in the same pathway (i.e., *DAXX* and *ATRX*) are needed for tumorigenesis to happen [41]. In one case, a somatic mutation of *PRKAR1A* was coexisting [29]. *TP53* mutations are associated with a high grade of genomic instability, they seem to occur at an early stage of tumorigenesis, and they are mutually exclusive with *USP8* mutations, thus shown to be driver mutations [29]. Tumors presenting with such mutations have also been described to be larger and more aggressive [38]. 

***BRAF*** is a well-known oncogene encoding a protein involved in the MAP-kinases downstream pathway. Somatic *BRAF* V600E mutation has been reported in several tumors; in 2018, such a mutation was detected in 16.5% of a case series of *USP8*-WT corticotrope adenomas; this alteration was not mutually exclusive with *USP48* mutations [42]. The suggested mechanism triggered by *BRAF* V600E mutation involves constitutive activation of BRAF and the MAP-kinases pathway, thus stimulating cell proliferation and, in corticotrope cells, ACTH synthesis and secretion. The increase in ACTH levels may be explained by a stimulation of the ERK signaling pathways [39] and an increase in the activity of transcriptional factors such as Nur77, c-Fos, and c-Jun, which induce *POMC* transcription in corticotrope cells [42].

A following case series, however, found such a mutation in a single case out of 94 examined adenomas [41], while other authors have not found any *BRAF* mutations, even with whole exome sequencing [8,9,39]. These case series also included a Chinese population, thus making this variability unlikely due to ethnic background only. Clarifying the real-world prevalence of the *BRAF* V600E mutation in corticotrope adenomas may be of great importance given the existence of targeted drugs already used in several types of cancer harboring the V600E mutation. In fact, Chen et al. presented preclinical evidence of the potential usefulness of the BRAF-inhibitor vemurafenib in *BRAF*-mutated primary corticotrope adenoma cells, showing a reduction in ACTH secretion with no difference in cell death rates [42]. More studies on the prevalence of *BRAF* mutations in Cushing’s disease are therefore needed to evaluate the possible use of this antisecretory effect in patients with a high surgical risk.

***NR3C1*** encodes the glucocorticoid receptor (GR), composed of a DNA binding domain, a ligand binding domain, and a transactivation domain. The GR is bound to HSP90 and immunophilins in the cytoplasm in its inactive form; in corticotrope cells, in the presence of glucocorticoids, the GR translocates to the nucleus and inhibits *POMC* expression and subsequent ACTH secretion [47]. Many other proteins modulate GR activity: Brg1 and HDAC2 contribute to *POMC* repression [48]; conversely, TR4 binds the GR and interferes with its binding with the *POMC* promoter [47]. Mutations of *NR3C1* have been described in glucocorticoid resistance syndrome in association with bilateral adrenal hyperplasia [49]; Next Genome Sequencing studies have then found rare loss-of-function *NR3C1* mutations in ACTH-secreting adenomas [50]. Such mutations cause truncated or non-functioning variants of the GR, with a reduced affinity to glucocorticoids and/or conformational alterations caused by structural instability, thus altering its responsiveness to glucocorticoids and/or its regulatory activity [50,51]. The described mutations are characterized by different molecular alterations in the corticotrope cells, and the reasons and consequences of this variability are yet to be completely understood [52].

Notably, these mutations are rarely found in corticotrope adenomas, with a reported prevalence of 6.2–6.3% of examined adenomas, whereas glucocorticoid resistance is one of the hallmarks of Cushing’s disease, implying that GR dysfunction has a pivotal role in its pathophysiology, regardless of *NR3C1* mutational status [38]. In fact, mutations in genes codifying for HSP90, TR4, CABLES1, and associated proteins have all been described in corticotrope adenomas and have been associated with features of glucocorticoid resistance [11], with some of the underlying molecular mechanisms yet to be demonstrated [53].

***CABLES1*** codifies for a tumor suppressor often altered in many different types of cancer [54]. CABLES1 inhibits CDK2 by connecting it to Wee1, and it stabilizes p21; these events contribute to cell cycle arrest. Notably, CABLES1 is regulated by Akt, which is part of the EGFR signaling pathway, linking EGF and cell cycle regulation and potentially giving CABLES1 a pivotal role in the pathophysiology of corticotrope adenomas. In AtT-20 cells, CABLES1 levels normally increase in response to glucocorticoids, together with many other molecular responses involving c-Myc, GADD45, and other proteins, contributing to the glucocorticoid-mediated negative feedback mechanisms on cell proliferation [53]. Moreover, the administration of a USP8-inhibitor in AtT-20 cells led to a suppression in proliferation and an increase in the CABLES1 levels and GADD45, possibly demonstrating a role for CABLES1 in the suppression of pathologic proliferation [55]. In fact, CABLES1 levels have been shown to decrease in ACTH-secreting adenomas [53,55]. The role of CABLES1, however, may be overestimated in AtT-20 cells; in fact, CABLES1-knockout mice showed normal pituitary development up to one year, suggesting that other mechanisms may be required for corticotrope adenoma formation [53].

Hernández-Ramírez et al. described four loss-of-function heterozygous *CABLES1* mutations among 181 patients affected by Cushing’s disease [56]. Despite the low number, given the clinical characteristics of the four described patients (i.e., age and tumor size), it is conceivable that *CABLES1* mutations may predispose pediatric patients to the formation of corticotrope adenomas [56].

***GNAS*** codifies for the Gsα subunit of G proteins involved in several receptor signal transduction via the cAMP pathway. Missense mosaic mutations of the R201 codon of this gene cause McCune–Albright syndrome (MAS). These gain-of-function modifications cause hyperactivation of Gsα and constitutive activation of the cAMP pathway. This translates into a clinical picture of polyostotic fibrous dysplasia, café-au-lait skin lesions, and hyperfunction of potentially all endocrine glands [57]. MAS patients can present with somatotroph or lactotroph pituitary adenomas, and with hypercortisolism secondary to adrenal hyperplasia or adenoma, but not ACTH-secreting adenomas [58]. To date, two somatic *GNAS* mutations affecting Q227 and R201 codons have been identified in corticotrope adenomas via DNA exome amplification and oligonucleotide probes or sequencing, without any clinical feature of MAS [59,60]. Interestingly, the same mutation described by Riminucci et al. was found in one of the 22 patients undergoing whole-exome sequencing by Chen et al. [42].

### 2.2. Evaluation of Somatic Mutations in Peculiar Settings

#### 2.2.1. Pituitary Carcinoma

Pituitary carcinoma is a very rare disease, and its pathogenesis is yet to be understood. This tumor has been reported to arise from biologically aggressive pre-existing adenomas [61]. Mutations involving *TP53* and *ATRX*, and potentially *MSH2* (in a patient with Lynch syndrome) have been described in this type of cancer [45,61,62,63]. Moreover, the mutational status of pituitary cancer cells may be altered by chemotherapy and allow for new therapeutic strategies: Lin et al. reported a case of pituitary carcinoma presenting as hypermutated after chemotherapy and responding to immunotherapy [64]. 

#### 2.2.2. Nelson’s Syndrome

This syndrome is characterized by the development of a pituitary ACTH-secreting adenoma after bilateral adrenalectomy. The analysis of these adenomas has shown alterations in *NR3C1* and *TP53* [61,65]. Pérez-Rivas et al. also found *USP8* mutations in 15 of 33 cases, with higher ACTH levels after surgery but without any other clinical differences compared to *USP8*-WT tumors [66].

#### 2.2.3. Silent Corticotrope Adenoma (SCA)

These corticotrope adenomas are associated with increased ACTH secretion but no hypercortisolism and clinical hallmarks of Cushing’s disease are present, making them similar to non-functioning pituitary adenomas [67]. SCAs may have a different origin and molecular pathogenesis than other corticotrope adenomas. The absence of hypercortisolism is not yet understood. Some authors have suggested that these tumors originate from pars intermedia POMC-positive cells, rather than from the anterior lobe. Other authors have reported the secretion of high-molecular-weight ACTH and alterations of prohormone convertase resulting in altered POMC cleavage [61]. Mouse models of SCAs and in vitro studies showed a reduced expression of tumor suppressor genes *RB1* and *KLK10*, and altered expression of other genes related to tumor progression and metastasis [68]. Moreover, transcriptome and proteome analysis comparing gene expression between SCAs and functioning corticotrope adenomas identified alterations in endoplasmic reticulum protein processing and in ACTH synthesis such as the overexpression of PCSK1N, an indirect inhibitor of ACTH maturation [69]. These differences may explain the altered ACTH secretion in SCAs [68,69,70,71]. Interestingly, *USP8* mutations seem to be much less frequent in SCAs, supporting the hypothesis of profound differences with Cushing’s disease adenomas [29,72]. SCAs also share more similar molecular alterations with ACTH-secreting macroadenomas than with ACTH-secreting microadenomas [73,74]. *HMGA* overexpression has been described in all histotypes of pituitary adenomas, with a higher prevalence for SCAs compared to corticotrope adenomas. HMGA dislocates HDAC2 from its binding with pRB, causing a un-inhibition of the E2F transcription factor, and it is also associated with an overexpression of *CCN2B* [13,75], possibly causing cell cycle dysregulation and increased cell growth. 

#### 2.2.4. Crooke’s Cell Adenomas

Crooke’s hyaline change consists of an excess of cytokeratin filaments causing an enlargement of corticotrope cells as a consequence of overexposure to exogenous or endogenous glucocorticoids. Crooke’s cell adenomas are characterized by >50% of cells showing Crooke’s change. This change usually impairs the corticotrope cell secretion of ACTH, so that clinical features of Cushing’s disease are absent, making these cases a variant of silent corticotrope adenomas. In some cases, however, Cushing’s disease develops in the presence of such adenomas. The distinctive pathophysiological mechanisms of these tumors are not yet understood, but an increased risk of invasiveness, recurrences, and transformation to metastatic carcinoma has been described [61,76]. A mutation of *SMO*, which codifies for a transmembrane GPCR involved in hedgehog signaling, has been described in one patient. SMO is deubiquitinated by USP8 and this modification alters its target region, possibly linking this mutation to the already described mechanisms of *USP8*-mutated adenomas [29]. 

### 2.3. Germline Mutations 

#### 2.3.1. Multiple Endocrine Neoplasia (MEN) 1

MEN1 syndrome is characterized by the variable association of parathyroid hyperplasia, gastroenteropancreatic NETs, and pituitary adenomas, and less frequently by tumors of other tissues, without a clear genotype–phenotype correlation [77]. MEN1 syndrome is caused by germline mutations of *MEN1*, encoding menin, which is a tumor suppressor gene involved in genome stability and cell cycle regulation. More than 1000 mutations have been described. Deletion or other loss-of-function mutations of the other allele cause a loss of heterozygosity and tumor development [78]. Corticotrope adenomas are rare in MEN1, representing 4.4–5% of all pituitary histotypes [78,79,80]; when considering proband patients presenting with a pituitary adenoma as the first sign of MEN1, this percentage arises to 15.4% [79], highlighting that corticotrope adenomas may be the first manifestation of this syndrome. This may be more evident among children: a retrospective analysis of 238 pediatric patients with Cushing’s disease found that six of them affected by MEN1 had ACTH-secreting adenoma as the first sign of the syndrome, suggesting careful evaluation of the familial history of pediatric patients presenting with Cushing’s disease, and subsequent testing if MEN1 is suspected [81].

#### 2.3.2. Multiple Endocrine Neoplasia (MEN) 2

MEN2A and MEN2B are caused by gain-of-function *RET* mutations, with a dysregulation of its tyrosine-kinase activity, involving RAS and RAF and subsequent alterations in cell survival and proliferation. These syndromes are characterized by the presence of medullary thyroid cancer and pheochromocytomas, associated with hyperparathyroidism in MEN2A and with ganglioneuromas and marfanoid habitus in MEN2B. Sometimes, ectopic ACTH secretion can be found [82], but as per corticotrope pituitary adenomas, only three cases of Cushing’s disease in patients affected by MEN2 have been reported so far, two in MEN2A [83,84] and one in MEN2B [85]. It is unclear whether these represent rare coincidences or germline *RET* mutations do have at least a permissive role in the formation of these adenomas. Interestingly, the two MEN2A cases were initially mistaken for MEN1, given the presence of pituitary adenoma and hyperparathyroidism, suggesting caution in interpreting a negative *MEN1* genetic testing in such a scenario [84].

#### 2.3.3. Multiple Endocrine Neoplasia (MEN) 4

MEN4 is an extremely rare MEN1-like syndrome caused by germline mutations of the *CDKN1B* gene [86], apparently associated with Cushing’s disease [87]. The transcript of this gene, p27/Kip1, has an inhibitory effect on cell cycle progression. This syndrome was recently defined, after a germline nonsense mutation had been described in a patient affected by acromegaly and hyperparathyroidism, and in three out of six of her relatives who underwent testing, in the absence of *MEN1* mutations [88]. The following year, a study found another inactivating mutation of the same gene in a patient with Cushing’s disease, small-cell neuroendocrine cervical carcinoma, and hyperparathyroidism [89]. Other studies have failed to find such mutations in patients with *MEN1*-WT MEN1-like syndrome, underlining the extreme rarity of these reported mutations [90,91]. In fact, a subsequent study searched and found CDK-inhibitors potential (but not certainly) pathogenic mutations in seven of 196 patients affected by MEN1-like syndrome: one of them carried a *CDKN1B* mutation and had no pituitary adenomas, while a patient and one of her relatives carrying a *CDKN1A*/p21 mutation were affected by a prolactinoma; none of these seven patients were affected by Cushing’s disease [92,93]. A more recent study, however, analyzed 211 mostly pediatric patients affected by Cushing’s disease via Next Generation Sequencing and found five of them (2.6%) carrying germline *CDKN1B* variants, three of which were pathogenic or likely pathogenic [94].

#### 2.3.4. Carney Complex 

Carney complex (CNC) usually presents with spotty skin pigmentation, myxomas, endocrine tumors, and Schwannomas. Hypercortisolism can be present, generally because of primary pigmented nodular adrenocortical disease (PPNAD), present in 2/3 of CNC patients. Growth hormone (GH)- and, less frequently, prolactin (PRL)-secreting pituitary adenomas have been reported. Inactivating mutations of the tumor suppressor *PRKAR1A* gene are the main cause of CNC, although other genetic alterations have been described [95].

In this setting, Cushing’s disease has been described in one adult and one pediatric patient carrying a germline *PRKAR1A* mutation; loss of heterozygosity was reported as the pathogenetic mechanism of adenoma formation [96,97].

#### 2.3.5. 3P Association (3PA)

Germline mutations in *SDHA*, *SDHB*, *SDHC*, and *SDHD* (collectively, SDHx) are associated with the development of PRL- and GH-secreting adenomas, pheochromocytomas, and/or paragangliomas, a syndrome named 3P association. One *SDHD* and one *SDHB* germline variant of uncertain significance were identified in five patients affected by sporadic cases of Cushing’s disease, who did not have any history or sign of pheochromocytoma or paraganglioma [98]. To date, no other reports or studies have explored the relationship between SDHx and corticotrope adenomas.

#### 2.3.6. USP8-Related Syndrome

The role of *USP8* in corticotrope adenomas has already been discussed above. As for germline mutations, however, in 2019, Cohen et al. [37] published a case report of a young female patient presenting with recurrent Cushing’s disease, developmental delay, dysmorphism, ichthyosiform hyperkeratosis, chronic lung disease, chronic kidney disease, hyperglycemia, dilated cardiomyopathy, heart failure, and history of hyperinsulinism and partial GH deficiency. Whole exome sequencing of DNA samples from the patient and three first-degree relatives revealed the presence of a de novo *USP8* germline mutation affecting the 14-3-3 binding region of the protein, resulting in a gain of function alike the mechanisms reviewed above. No other similar cases have been reported yet. 

#### 2.3.7. DICER1

DICER1 is a class III cytoplasmic endoribonuclease that cleaves double-stranded precursor RNAs into siRNAs and miRNAs that interfere with the target RNA translation. Germline *DICER1* mutations cause a defect of this physiologic gene silencing process, resulting in an extremely rare pediatric tumor syndrome. A typical and severe manifestation of this syndrome is pituitary blastoma, presenting with ACTH-dependent hypercortisolism [99]. In an in-depth study by de Kock et al., germline *DICER1* mutations were found in nine children affected by pituitary blastoma, with the other allele presenting somatic non-synonymous mutations (six cases) or loss of heterozygosity (one case) in the tumoral tissue, suggesting a potential two-hit model for the tumorigenesis of pituitary blastomas in DICER1 syndrome [100]. Another study by Sahakitrungruang et al. identified a somatic and a germline *DICER1* mutation in the DNA of a pituitary blastoma, supporting such a model [101]. Notably, it has recently been suggested that DICER1 variants may be associated with an increased risk of sporadic corticotrope adenomas, regardless of DICER1 syndrome, although no data are available to assess the pathogenic meaning of these variants [102].

#### 2.3.8. Lynch Syndrome

Lynch syndrome is caused by mutations affecting the mismatch repair pathway, with a subsequent increased risk of several types of cancer. Two cases of Cushing’s disease have been reported to date: one adenoma and one carcinoma. Interestingly, the adenoma showed homozygous somatic *MEN1* mutations, possibly as a consequence of the increased risk of genetic alterations in the context of this syndrome [63,103]. 

#### 2.3.9. Beckwith–Wiedemann Syndrome (BWS)

This syndrome is caused by imprinting alterations of the 11p15 region, containing genes encoding for proteins involved in somatic growth and cell cycle regulation. A phenotype correlation with the various epigenetic alterations has been described, with a variably increased risk of developing embryonal and other tumors. Brioude et al. published a case report of a *USP8*-mutated corticotrope adenoma arising in a patient with an incomplete presentation of BWS, interestingly showing an association between epigenetic and genetic alterations [104]. 

#### 2.3.10. Tuberous Sclerosis Complex (TSC)

Germline mutations of *TSC1*/hamartin and *TSC2*/tuberin are responsible for TSC. The two proteins encoded by these genes form a dimer, acting as a tumor suppressor via the inhibition of the mTOR pathway. A dysregulation of cell growth and proliferation causes the formation of multiple hamartomas in various organs, cognitive impairment, and epilepsy [105]. Endocrine dysfunction can arise, and Cushing’s disease has been reported in two patients affected by TSC [106,107]. No other associations have been reported to date.

#### 2.3.11. Non-Syndromic Germline Mutations: Familial Isolated Pituitary Adenoma (FIPA) and CDH23

**FIPAs** (familial isolated pituitary adenomas) are defined as pituitary adenomas occurring in at least two members of a single family, or at an early age, in the absence of the other syndromic features described above. Mutations in *AIP* gene, encoding a co-chaperone protein with multiple targets, have been associated with mostly somatotroph and lactotroph adenomas, and screening for these mutations is recommended in this setting [87]. Rare cases of the detection of such mutations in pediatric or young adult patients affected by Cushing’s disease have also been reported [108,109], especially the mutation R16H, which has an uncertain pathogenetic significance [110]. However, only 20% of patients presenting with FIPA show *AIP* mutations, demonstrating the presence of other genetic alterations yet to be discovered. A single study reported the presence of *MEN1* mutation in three patients from two families of their cohort, one of which had a corticotrope adenoma and none of them with other features of MEN1 syndrome, suggesting a potential role of *MEN1* in *AIP*-WT FIPAs [111]. It is of note, however, that MEN1 syndrome (described above) is characterized by metachronous tumors, so that isolated pituitary adenomas in *MEN1* germline mutation carriers may represent the first occurring neoplasia of this syndrome and not a clinical picture of FIPA.

***CDH23*** encodes for a calcium dependent intercellular adhesion glycoprotein involved in Wnt pathway regulation. Homozygous mutations of this gene have been associated with Usher syndrome, characterized by congenital sensorineural hearing loss and later retinitis pigmentosa. Four germline mutations in four different sporadic ACTH-secreting adenomas, one of which is homozygous, have been reported in a single study and need further analysis. No association between Usher syndrome and corticotrope adenomas has been reported [87,112].

## 3. Conclusions

In this review, we presented the most recent somatic and germline variants underlying ACTH-secreting adenomas that are involved in tumor development and progression. Several alterations have been reported, with substantial advances in our understanding of the molecular pathogenesis and pathophysiology of Cushing’s disease. In the future, both basic research and clinical studies are needed to deepen our knowledge of the molecular basis of this disease as the different described mutations may have important prognostic implications. First, the association between these mutations and several clinical features (such as adenoma size, levels of ACTH secretion, local invasiveness) need to be thoroughly investigated to clarify whether the mutational status of these genes correlate with different prognosis in terms of the response to treatment and risk of recurrences. Second, further research is needed on the diagnostic application of the reported mutations (e.g., exploring their histological usefulness). Last but not least, new applications of targeted drugs (e.g., multi kinase inhibitors, agnostic drugs) need to be explored, and new targeted drugs (such as USP8-inhibitors) may be studied to offer new therapeutic scenarios for patients (e.g., inoperable patients or patients at high risk of recurrences). Nonetheless, intriguing murine and in vitro research has been carried out, but preclinical research is still lacking and no studies on humans have been published to date, making these scenarios not yet foreseeable.

## Data Availability

Not applicable.
